# Interactions of small molecules with DNA junctions

**DOI:** 10.1093/nar/gkac1043

**Published:** 2022-11-16

**Authors:** Kane T McQuaid, Angélique Pipier, Christine J Cardin, David Monchaud

**Affiliations:** Department of Chemistry, University of Reading, Whiteknights, Reading RG6 6AD, UK; Institut de Chimie Moléculaire de l’Université de Bourgogne (ICMUB), CNRS UMR 6302, UBFC Dijon, 21078 Dijon, France; Department of Chemistry, University of Reading, Whiteknights, Reading RG6 6AD, UK; Institut de Chimie Moléculaire de l’Université de Bourgogne (ICMUB), CNRS UMR 6302, UBFC Dijon, 21078 Dijon, France

## Abstract

The four natural DNA bases (A, T, G and C) associate in base pairs (A=T and G≡C), allowing the attached DNA strands to assemble into the canonical double helix of DNA (or duplex-DNA, also known as B-DNA). The intrinsic supramolecular properties of nucleobases make other associations possible (such as base triplets or quartets), which thus translates into a diversity of DNA structures beyond B-DNA. To date, the alphabet of DNA structures is ripe with approximately 20 letters (from A- to Z-DNA); however, only a few of them are being considered as key players in cell biology and, by extension, valuable targets for chemical biology intervention. In the present review, we summarise what is known about alternative DNA structures (what are they? When, where and how do they fold?) and proceed to discuss further about those considered nowadays as valuable therapeutic targets. We discuss in more detail the molecular tools (ligands) that have been recently developed to target these structures, particularly the three- and four-way DNA junctions, in order to intervene in the biological processes where they are involved. This new and stimulating chemical biology playground allows for devising innovative strategies to fight against genetic diseases.

## INTRODUCTION

The DNA alphabet is naturally restricted to four letters, i.e. A for adenine, C for cytosine, G for guanine and T for thymine (1,2) (although some creative scientists have achieved the juggling act to expand it to 6 in living semisynthetic organisms (3,4), and then 8 (5), mimicking what was discovered in bacteriophages >4 decades ago) (6–9). In contrast, the DNA structure alphabet is far richer, with >20 letters used to date as descriptors of secondary structures (10). The canonical, so called Watson–Crick, structure is referred to as B-DNA (as the X-ray crystallographic structure was obtained by Rosalind Franklin after hydration of a first sample (‘A’) of high-quality DNA provided by Rudolf Signer to Maurice Wilkins, consequently termed ‘B’) (11–14). Every other structure has consequently been categorized as a non-B-DNA structure, spanning from A-DNA (thus, dehydrated duplex) (11) to Z-DNA (duplex of inverted helicity, Z for zigzag) (10,15).

**Figure 1.**
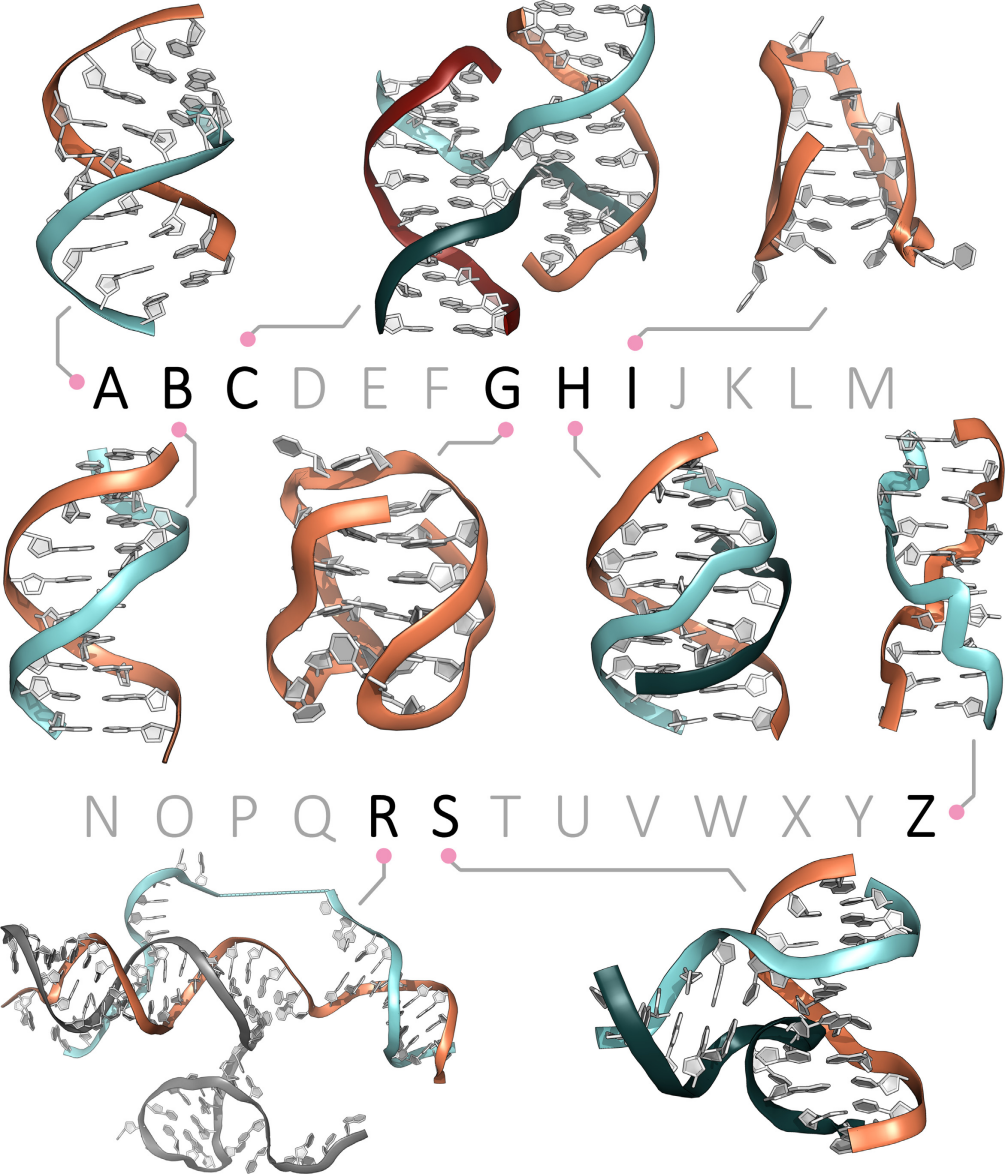
The DNA alphabet including the canonical B-DNA (or Watson–Crick duplex) along with a series of non-canonical structures including A-DNA (dehydrated duplex DNA), C-DNA (cruciform DNA, or four-way DNA junction), G-DNA (G-quadruplex DNA), H-DNA (for hinged DNA, or triplex DNA), I-DNA (i-motif), R-DNA (R-loop), S-DNA (for slipped DNA, or three-way DNA junction) and Z-DNA (for zigzag DNA).

The central dogma of biology, heralded by Francis Crick in 1957 (16,17), has placed the B-DNA at the very centre of all molecular biology efforts invested, and discoveries made, after the elucidation of its double helix structure (2,13,18). This has kept the limelight away from reports published in succeeding years on the ability of DNA to fold into a variety of non-B-DNA structures (Figure 1), including: the triple helix (or triplex (19), termed H-DNA given its homopurine (hPu)/homopyrimidine (hPy) nature, also referring to hinged DNA) (20), first identified in RNA in 1957 (21) before being characterized in DNA in 1979; (22) the G-quartet in 1962 (23), the constitutive unit of the quadruple helix G-quadruplex (G4-DNA, or G-DNA) whose formation was demonstrated in 1988; (24) the tetra-stranded four-way DNA junction proposed as a model to explain gene conversion in 1964 by Robin Holliday (consequently called the Holliday junction) (25), predicted in 1966 (26) and demonstrated *in vitro* (and termed cruciform DNA, C-DNA) in the early 80s; (27,28) the Z-DNA first detected in 1967 (as a B-DNA of inverted, left-handed helicity) (29) before being firmly confirmed in 1979 (30), etc.

The topological diversity of DNA stems from supramolecular chemistry considerations: nucleobases (A, C, G, T) associate through the formation of hydrogen bonds (H-bonds), two in the A=T base pair, three in the G$ \equiv$C base pair, allowing for a dynamic assembly/disassembly without substantial energy penalty. However, nucleobases are not exclusive in their H-bond-mediated association, and >20 different pairing modes are possible involving two of the four letters (31), offering a first degree of topological diversity (base pairing). Also, nucleobases can donate/accept H-bonds through both their Watson–Crick (2) and Hoogsteen (32,33) faces, which are not mutually exclusive and thus permit the formation of base triplets (34) and quartets (23), offering a second degree of topological diversity (strandness). On the basis of the above, the structural pluralism of nucleic acids is anything but surprising.

**Figure 2.**
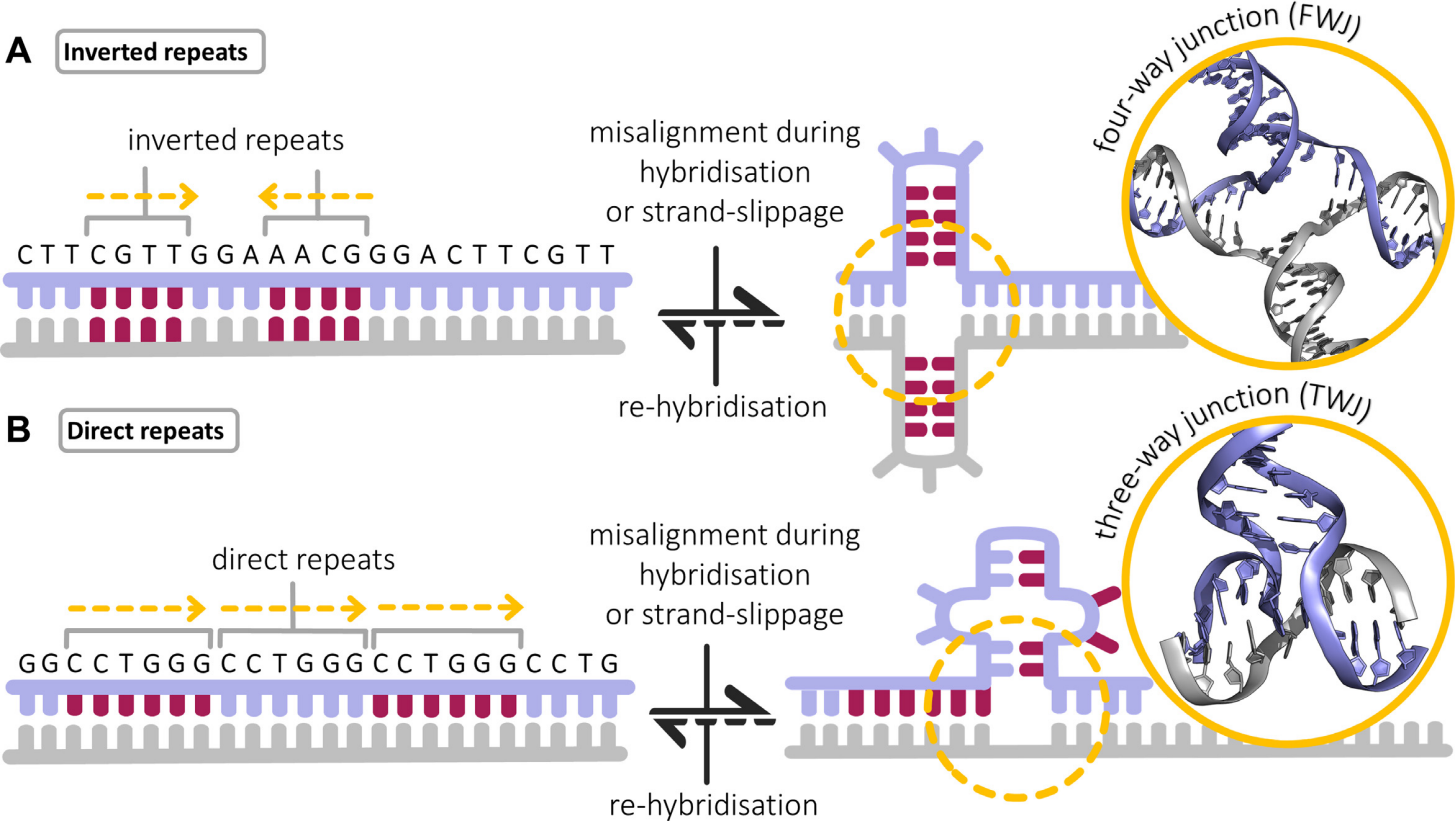
Schematic representation of junction-forming sequences, i.e. inverted repeats that can give rise to a four-way DNA junction (**A**) or direct repeats that can fold into a three-way DNA junction (**B**).

The reason why these alternative structures have been overlooked for years is that their existence in a cellular context is challenged by the chromatin environment, in which the dense DNA packaging massively favours B-DNA. This has made the demonstration of their existence in *in vivo*-like conditions daunting, and the chemical biology tools required to do so have reached the necessary degree of maturity only recently (e.g. the visualisation of G4s in fixed cells by the antibody BG4 in 2013 (35), in live cells by the small molecule SiR-PyPDS in 2020) (36). Also, the rules that dictate their formation in the genome are poorly understood even if the sequences these structures fold from (i.e. repeated sequences) are known to be both abundant and widespread in the human genome (repetitive DNA elements cover roughly half of our genome) (37,38). Non-B-DNA-prone sequences can be inverted repeats (IR, involved in the formation of four-way DNA junction (FWJ), or cruciform DNA, Figures 1 and 2A), direct repeats (DR, involved in the formation of three-way DNA junction (TWJ), also termed slipped-strand DNA, slipped DNA or S-DNA (39), Figures 1 and 2B), mirror repeats (MR, involved in the formation of H-DNA) (38,40,41), or short tandem repeats (STR, involved in the formation of hairpin DNA, G4-DNA and i-motif (iM or I-DNA, Figure 1) depending of the repeated motif) (42). Thanks to recent computational analyses of an entirely sequenced human genome, the abundance of the non-B-DNA-prone sequences can be calculated: it is established that the least abundant motif is MR, with ca. 70 000 occurrences (ca. 2/100 kb), while the most abundant motif, IR, has >6 000 000 occurrences (ca. 206/100 kb) (41). However, despite the abundance of sequences that could give rise to non-B-DNA structures, the timing and kinetics of their folding are still poorly understood. Also, this folding requires the involved sequences to be freed from the duplex constraint (i.e. open chromatin, damaged DNA), their formation is thus transient only and subjected to a permanent and careful surveillance by *ad hoc* enzymes (e.g. helicases) (43), which make their formation even less likely. This thus explains why non-B-DNA structures have not been considered as reliable genetic elements (and targets) for years. Despite all these constraints, some of these structures (G4s, iMs) have been isolated and identified by sequencing methods. Efforts invested *in vitro* (that is, with purified DNA fragments) confirmed their widespread formation in the human genome (with >500 000 G4- (44) and iM-forming (45) sequences), but not only (46) (e.g. >25 000 G4- (47) and iM-forming (48) sequences in plants) (49). Investigations performed *in vivo* distinctly highlighted the suppressive role of chromatin for non-B-DNA structure formation as only ca. 1% of the G4s detected *in vitro* (G4-seq) (44) were detected *in vivo* (G4 ChIP-seq) (50). More globally, a combination of footprinting experiments (KMnO_4_/S1 nuclease) and genome-wide sequencing (ssDNA-seq) allowed to link ssDNA regions in mammalian cells with non-B-DNA structures (C-, G-, H- and Z-DNA, ca. 20 000 motifs each) and then, these structures with regions involved in gene expression regulation (51). Further combining nuclease cleavage (S1 and P1) and sequencing (S1-END-seq and P1-END-seq, respectively) revealed that C- and H-DNA formation (at dinucleotide (TA)_*n*_ repeats and hPu/hPy repeats (e.g. (GAA)_*n*_), respectively) is both widespread in human cells but also strongly dependent on the cell status (cancerous versus non-cancerous cells) (52,53). These results, beyond confirming the existence on non-B-DNA structures *in vivo*, provide also a strong correlation between structure-prone sequences and both mutability and genetic instability (52) (often referred to as to RIM, for repeat-induced mutagenesis) (54,55). Indeed, their widespread and nonuniform distribution across our genome is significantly enriched in regulatory regions, and the distribution quite often overlaps with that of reported hotspots, i.e. regions prone to undergo breakage, deletions and translocations (38,41,55,56). This explains why repeated sequences are carefully patrolled and tightly controlled by genome surveillance systems, notably the DNA damage response (DDR) machinery (57–59).

As indicated above, to adopt higher-order structures, these sequences must be relieved from their duplex constraint and reannealed with misalignment or slippage. Their folding is thus dependent on, and coupled with, DNA transactions (transcription, replication) and repair. This makes them interesting targets for therapeutic approaches aimed at inflicting damage to highly active, that is, rapidly dividing cells. Indeed, once folded, these thermodynamically stable structures might act as roadblocks to polymerases, pausing and/or stalling their processivity, which is recognized as a situation of crisis (DNA damage) (60–63). It is therefore unsurprising that chemical biologists soon envisioned an original strategy in which the transient stabilization of non-B-DNA structures by small molecules (so called ligands) could be exploited to foster this situation of crisis notably in rapidly dividing, that is, cancer cells (62,64). The relevance of this approach is further substantiated by the fact that cancer cells are generally DDR-impaired (57), which makes them more sensitive to DNA damage-inducing agents than healthy cells. This explains why many of the therapeutics currently used in the clinic damage DNA, although through different modalities (65). However, these therapeutics target B-DNA (or their associated proteins such as topoisomerases) but none of them target non-B-DNA structures. This is clearly the major caveat of this field, which deserves to be addressed soon in order to lend credence to its strategic relevance. The most advanced molecule, the G4-stabilizer CX-5461 is currently in phase I (clinicaltrials.gov NCT02719977) (66) against advanced solid tumours (its parent molecule CX-3543, also known as Quarfloxin, was stopped after phase I). Without a successful example of non-B-DNA targeting agent in the clinic, this field will keep on suffering from a lack of legitimacy. Yet, in contrary to B-DNA, which offers poorly defined binding sites only (i.e. minor and major grooves, intercalation in between 2 bp), non-B-DNA structures offer a broad variety of structurally well-defined ligand binding sites, which makes highly selective targeting with small molecules possible. Chief among them are the G4-DNA (67–69), which display two accessible G-quartets surrounded by flexible loops, and DNA junctions (70–72), in which the junction point between the duplex arms (3 duplex arms for TWJ; four arms for FWJ) creates a cavity prone to welcome small molecules (*vide infra*).

The recent developments described in this review will be focused on the targeting of DNA junctions, as that of G4-DNA is regularly covered by authoritative reviews (68,69,73–77) to which interested readers are invited to refer. These developments offer this field a shining message of hope, as these new ligands allow for a specific targeting of DNA junctions (78), yet in *in vitro* and cell-based assays only to date, which bears significant potential for delivering soon a new generation non-B-DNA targeting therapeutics for which the demonstration of clinical efficacy is greatly expected.

## STRUCTURE, FUNCTIONS AND TARGETING OF DNA JUNCTIONS

### The early days of DNA junction targeting

**Figure 3.**
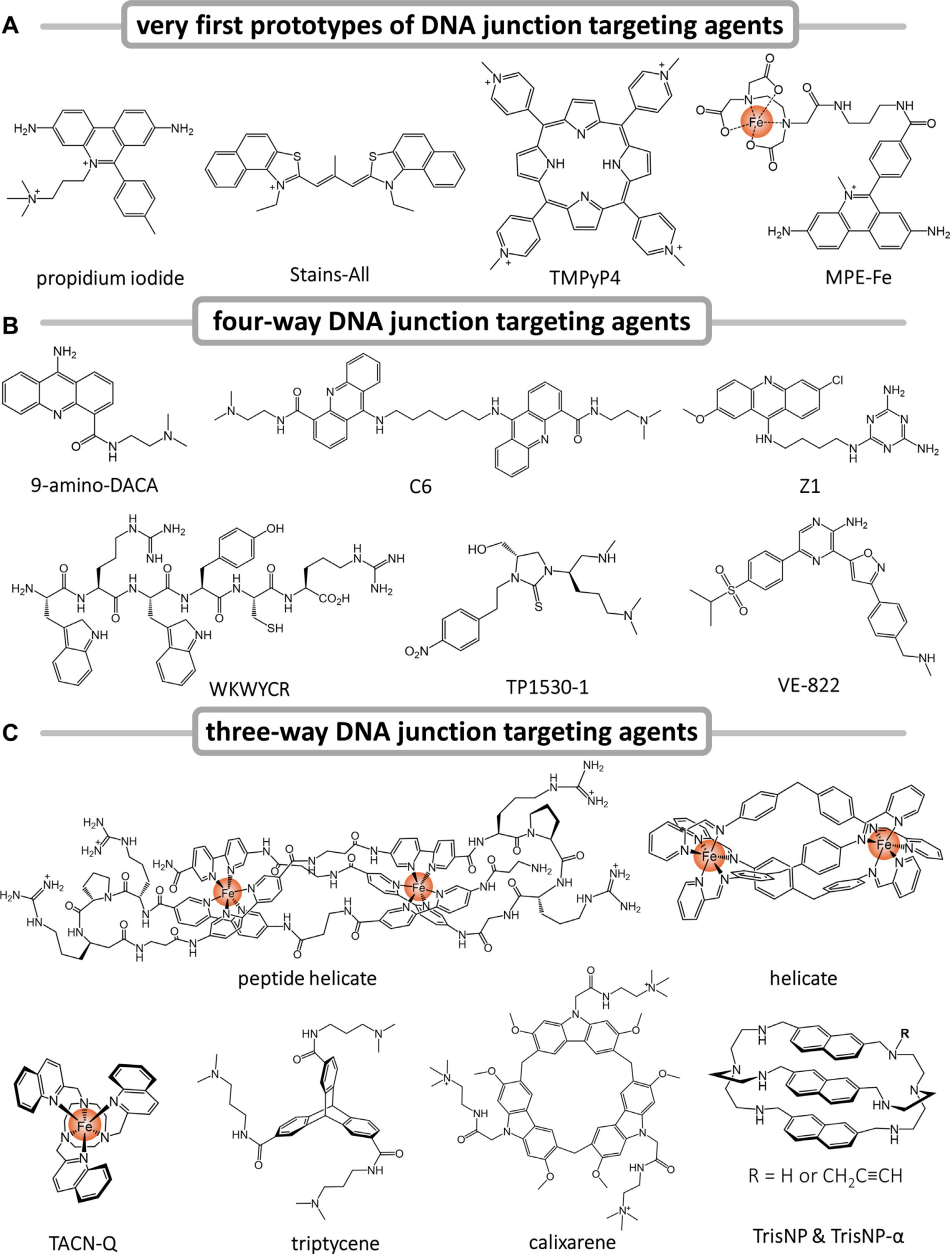
(**A**) Chemical structures of the very first DNA junction targeting agents used by Kallenbach *et al.* (79), i.e. propidium iodide, Stains-all, TMPyP4 and MPE-Fe (counter-ions are removed for clarity). (**B**) FWJ ligands used for either *in vitro* studies, i.e. 9-amino-DACA (115), C6 (118) and Z1 (122), or for cell-based assays, i.e. WKWYCR (132), TPI1530-1 (143) and VE-822 (146). (**C**) TWJ ligands including diiron helicate (171) and peptide helicate (177), the poly-aza-macrocycle TACN-Q (178) along with a triptycene (181), a calixarene (186), and two azacryptands TrisNP and TrisNP-α (156).

The field of non-B-DNA structures and related ligands was undoubtedly pioneered by Neville Kallenbach (72,79). In a series of articles published in the mid 80’s-mid 90’s, he provided both biophysical characterizations (e.g. by calorimetry (80), gel electrophoresis (81), chemical footprinting (82,83), etc.) of DNA junctions (but also of G4-DNA) (84,85) and the first insights into how small molecules (Figure 3A), mostly fluorescent dyes, i.e. the DNA labelling agent propidium iodide (86), the cyanine stains-all (87), the porphyrin TMPyP4 (88), but also cleaving agents such as methidium-propyl-EDTA-Fe(II) (MPE-Fe) (89), interact with them (and with G4s) (90). Of course, these studies were limited to *in vitro* investigations and the selectivity for DNA junctions over B-DNA, of utmost importance for chemical biology and medicinal chemistry, was not investigated. Anyway, these studies were instrumental in that they spurred on research aiming at ultimately investigating the cellular effects on these new genetic targets and molecular tools (further discussed below).

### Targeting four-way DNA junctions

**Figure 4.**
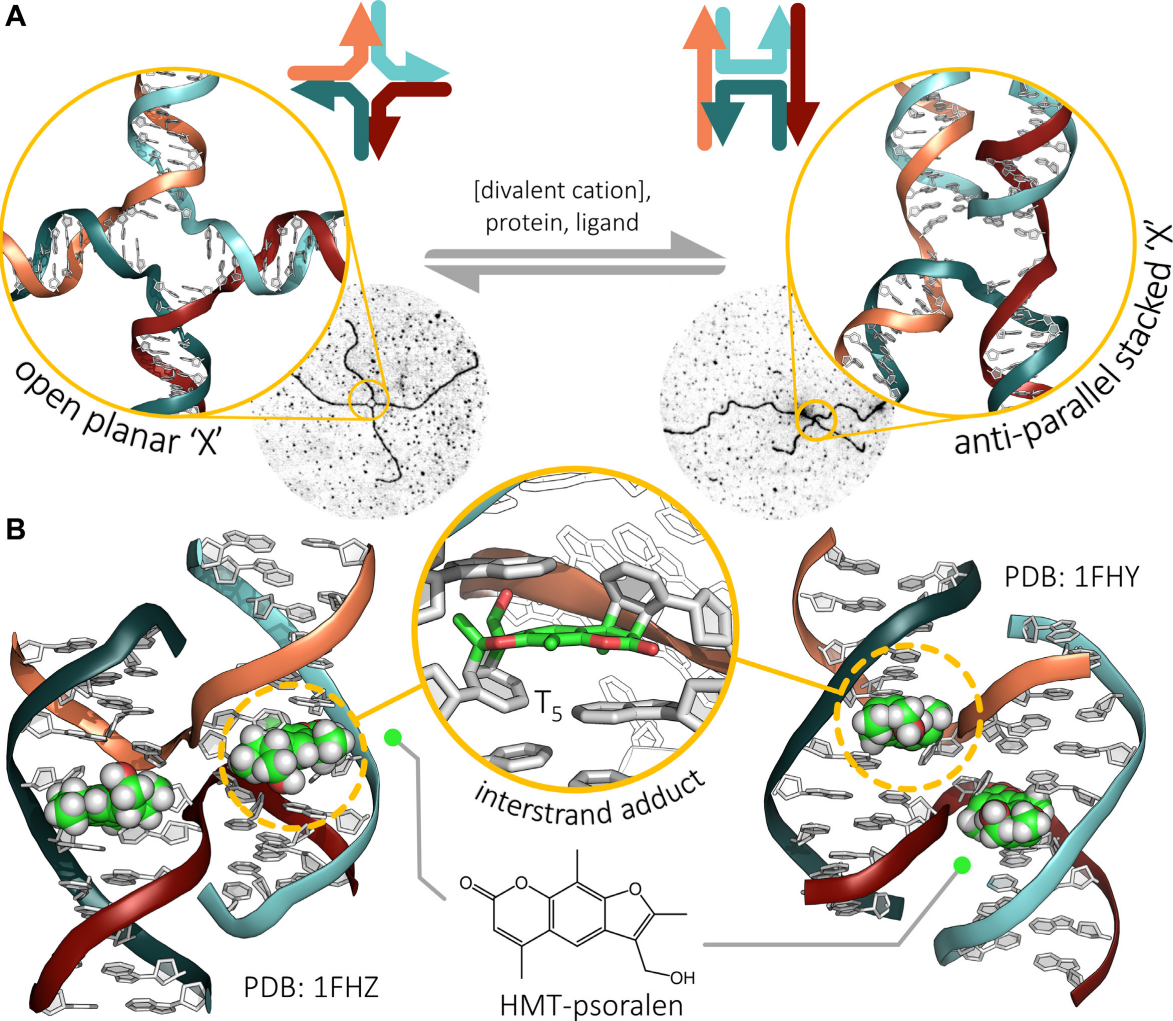
(**A**) The conformational plurality of a four-way DNA junction (‘open’ versus ‘stacked’ junction) that can be modulated by external mediators including cations (e.g. Mg^2+^), enzymes (e.g. RuvA or Cre) and ligands (e.g. the peptide WKHYNY). SEM images from (103). (**B**) X-ray crystal structures (PDB: 1FHZ and 1FHY) (113) showing that the photochemical reaction of psoralen with the d(CCGCTAGCGG) sequence led to a stable four-way DNA junction (1FHY), whereas the native sequence crystallises as a B-DNA. The sequence d(CCGGTACCGG) gives a four-way junction both in native conditions and after reaction with the psoralen (1FHZ).

As indicated above, the existence of a four-stranded DNA structure was postulated by Robin Holliday in 1964 as an intermediate in homologous recombination (HR) (25,91). The possible formation of a central four-way junction intermediate (a ‘chiasma’) had already been discussed by Joseph G. Gall in 1954 (92), as a basis for the crossing-over mechanism, i.e. the reciprocal exchange of segments along pairs of homologous chromosomes. However, heroic efforts were needed to obtain a structural confirmation of the FWJ organization of this intermediate: various biochemical and physicochemical methods were implemented, including gel electrophoresis (93–95), fluorescence energy transfer (FET (96,97), then reported as FRET (98,99), for fluorescence resonance energy transfer), birefringence decay (100), and nuclear magnetic resonance (NMR) (101,102), along with some imaging techniques such as scanning electron microscopy (SEM) (103) and atomic force measurements (AFM) (104). Among these techniques, X-ray crystallography played a central role: (105) solid state analyses were indeed instrumental to solve the structure of FWJ, alone (106,107) or in interaction with enzymes in charge of the recombination (the resolvase RuvA (108,109) or the recombinase Cre (110), *vide infra*) (111) and then, small molecules (*vide infra*). All these techniques concurred in demonstrating the organisation of the FWJ, with its four arms that either extend from opposite sides of the central cavity, which is therefore wide open (the so-called open planar X-structure), or stack on each other two by two (the so-called stacked X-structure), which consequently closes the central cavity (Figure 4A) (70).

The initial junction structure was obtained while studying DNA mismatches, and a later study revealed that, in the crystal structure, junction formation is sequence dependent (112). These authors carried out a systematic study of the decamer sequences CC*n_1_n_2_n_3_N_1_N_2_N_3_*GG, where *N* can be any of the four common nucleotides, and *n* are specified accordingly to maintain the IR motif and thus self-complementarity of the sequences. From the 64 possible combinations, they concluded that the d(CCGGTACCGG) sequence gave the junction form under all conditions tested, whereas other sequences could be in equilibrium with A- or B-DNA forms. A way to control the conformation is the covalent binding of psoralen: (113) the closely related sequence d(CCGCTAGCGG) was found to crystallise as a B-form duplex, but the binding of psoralen after laser irradiation at the TA/TA step led to the stabilisation of the junction (Figure 4B). More generally, the X-ray structures obtained in presence of enzymes and ligands showed that these effectors can modulate the shape of the FWJ in order to structurally optimise the ligand/DNA association (induced-fit).

**Figure 5.**
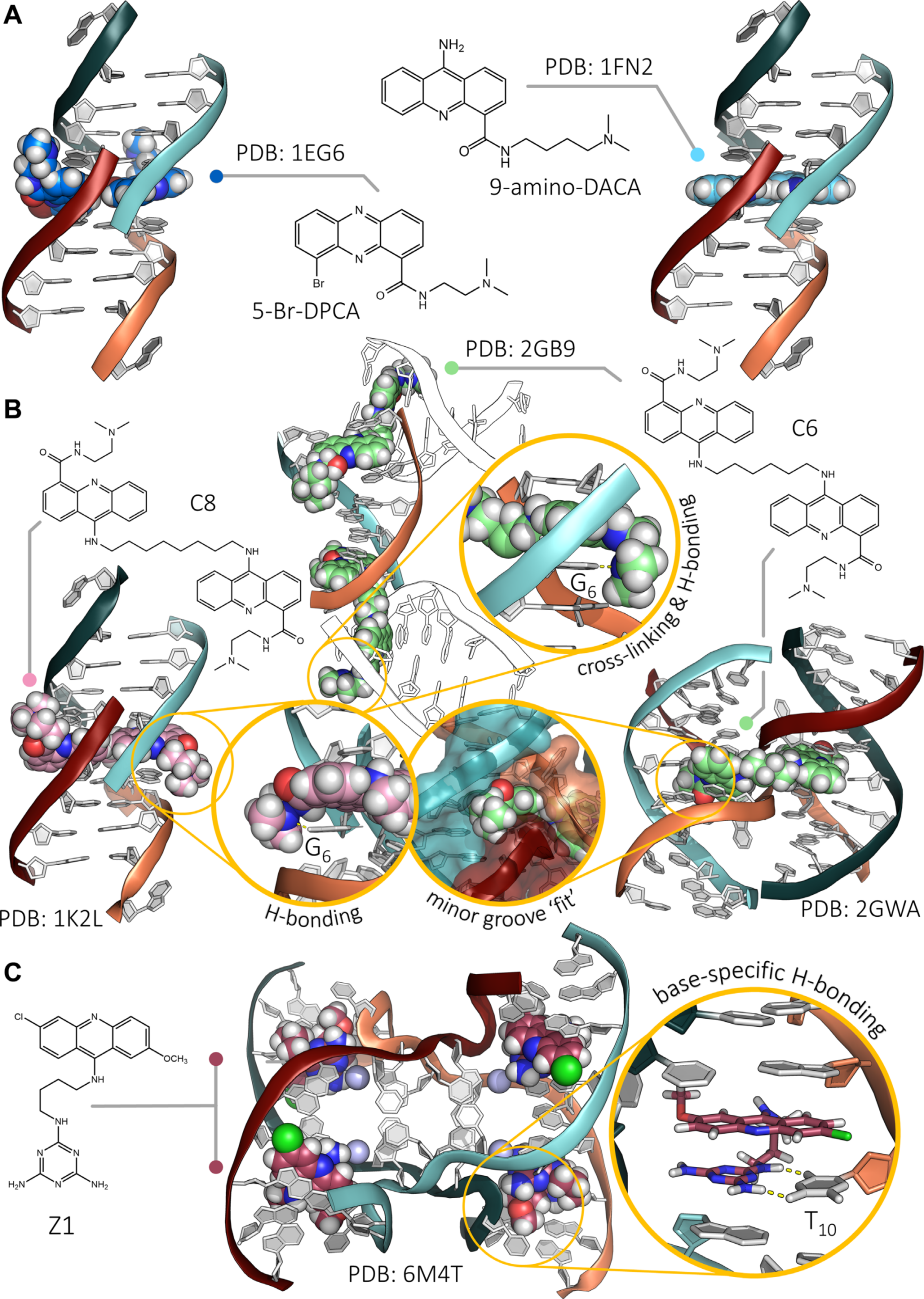
(**A**) X-ray crystal structures (PDB: 1EG6 (115) and 1FN2 (114)) of quasi-junction cavities obtained with the d(GCTACG) sequence in interaction with phenazine or acridine derivatives. (**B**) X-ray crystal structures (PDB: 1K2L (116), 2GB9 (117) and 2GWA (118)) of DNA junction in interaction with acridine dimers. (**C**) X-ray crystal structure (PDB: 6M4T) of a DNA junction in interaction with the triazine Z1 (122). The nucleobases involved in ligand/FWJ interactions are identified in the zooms.

Regarding the way small molecules interact with FWJs (Figure 3B), the first insights were obtained through the ligand-induced dimerisation of a short, 6-nucleotide (6-nt) duplex. In this study (114), incubation of the self-complementary d(CGTACG) sequence, or the closely related d(CG[5-BrU]ACG), with acridines (e.g. DACA, 9-amino-DACA, Figures 3B and 5A) (114) and phenazine (115) derivatives provided an asymmetric unit in which the terminal C of one strand invades the second helix at the ligand binding site, therefore creating a cavity in which two acridines nestle in between four base pairs (Figure 5A). We thus designed dimeric derivatives of DACA (linked through 1,6-diaminohexane or 1,8-diaminooctane linkers, named C6 and C8, respectively, Figures 3B and 5B) as possible bis-intercalators and determined two types of X-ray structures with d(CGTACG): a structure in which, behaving similarly to its monomeric counterpart (see above), C8 induces a FWJ-like assembly on the basis of a terminal C exchange and creates a H-bond with the residue G_6_ (Figure 5B); (116) and a structure in which C6 crosslinks two separate duplexes, each of its acridine units being intercalated in between two CG base pairs of a given duplex (Figure 5B) (117). Finally, the X-ray structure of C6 in interaction with a full FWJ resulting from the self-assembly of the d(CCGGTACCGG) sequence was successfully solved and refined (Figure 5B) (118). This clearly defined binding mode affirmed the suitability of the central cavity of FWJs to accommodate close contacts with dedicated ligands. In this example, the acridine units cause two A residues (A_6_) at the central TA step of the crossover strands to flip out to generate the ligand binding site in which both a long H-bond (between the DACA sidechain and the residue C_7_) and stacking interactions (in between a T and a C) with surrounding nucleobases are created. Later, it was demonstrated that DACA derivatives were also able to induce the formation of FWJs (119); however, these studies, though elegant and insightful, were limited to structural investigations only, owing to the known tendency of acridine derivatives to intercalate within duplexes (120). Their use in cell-based assays would have been troublesome because of their strong B-DNA interaction, which would have blurred the relevance of FWJs as therapeutic target: indeed, it would have been impossible to discriminate the origins of the cellular effects observed between those originating in B-DNA intercalation (the most likely event) and those originating in FWJ interaction (the less likely event). This is mostly due to the fact that these ligands do not target the unique structural component of FWJs (i.e. the central cavity), but instead bind to the duplex arms at the proximity of the cavity, which makes specific interaction unattainable. One possibility would have been to structurally fine-tune acridines to preclude intercalation in a manner reminiscent of what was done with G4 ligands, which led to the design of the 3,6,9-trisubstituted acridine BRACO-19 (121). Another possibility was to target the central cavity of FWJs; as discussed further below, this was done with a series of short peptides but without firm structural characterization of their FWJ binding mode.

As with the original Holliday junction structure, which was a serendipitous discovery while studying DNA mismatches, a recent mismatch study also uncovered junction formation (122). A potent DNA-binding compound triaminotriazine-acridine conjugate (Z1, Figure 3B) functions by targeting T:T mismatches in CTG trinucleotide repeats that are responsible for neurological diseases such as myotonic dystrophy type 1, but its binding mechanism remains unclear. The crystal structure of this ligand was solved in a complex with DNA containing three consecutive CTG repeats with three T:T mismatches. Surprisingly, direct intercalation of two Z1 molecules at both ends of the CTG repeats induced T base flipping (T_4_), a H-bond between Z1 and a T (T_10_) and DNA backbone deformation to form a four-way junction (Figure 5C). The core of the complex unexpectedly adopts a U-shaped head-to-head topology to form a crossover of each chain at the junction site. The crossover junction is held together by two stacked G:C pairs at the central core that rotate with respect to each other in an X-shape to form two nonplanar minor-groove-aligned G:C:G:C tetrads. Two stacked G:C pairs on both sides of the central core are involved in the formation of pseudo-continuous duplex DNA. However, and again, these studies were limited to structural investigations only owing to the lack of information concerning the specificity of Z1 for FWJs that, combined with the known health-threatening nature of triazines (notably widely used as herbicides) (123), is not the best guarantee of a bright future for this series of compounds.

### The relevance of FWJ in cells

**Figure 6.**
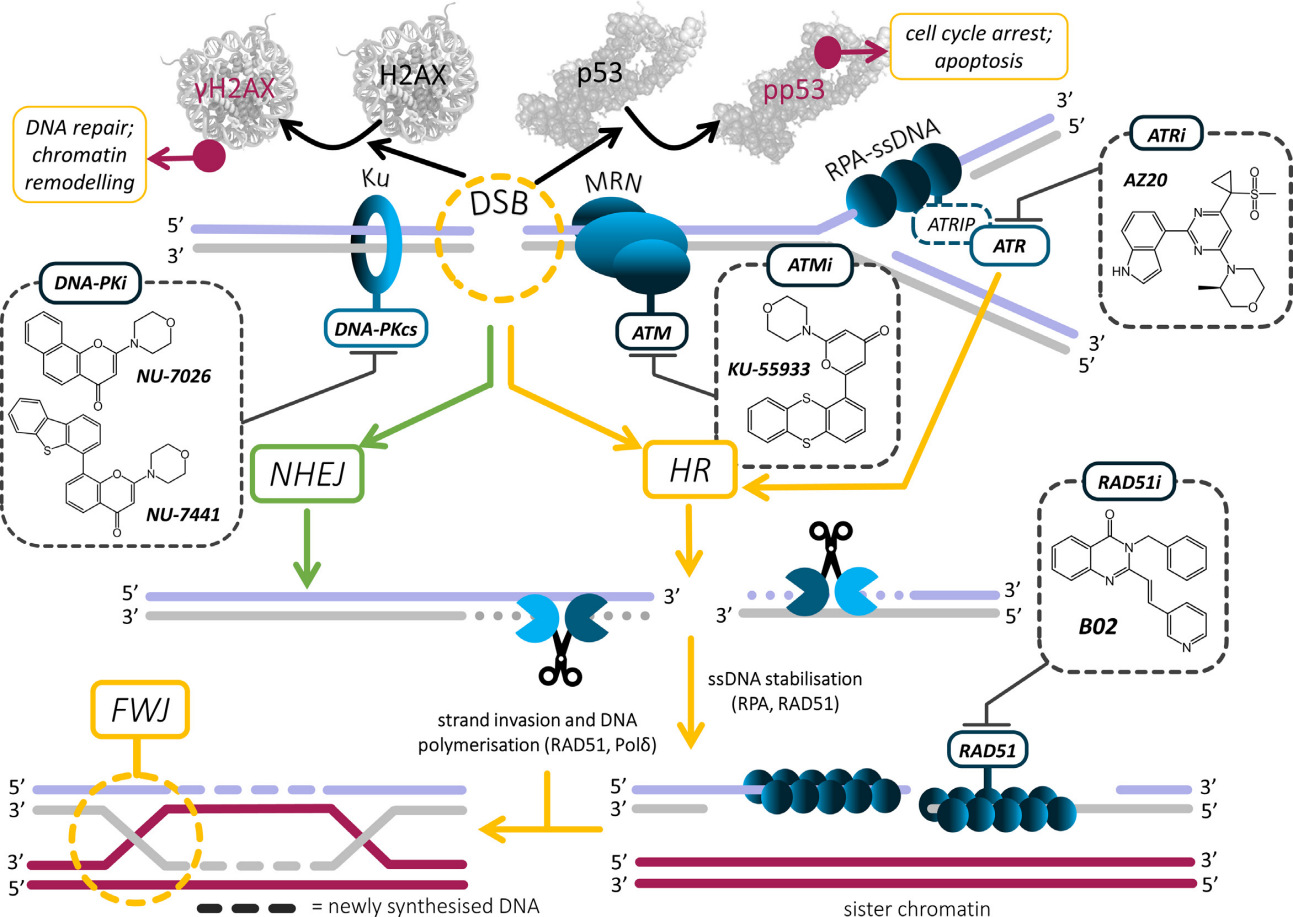
Double-strand breaks (DSBs) and stalled replication forks (RFs) are repaired by homologous recombination (HR) and non-homologous end joining (NHEJ) mechanisms, mediated by DNA-PK, ATM and ATR kinases, and RAD51. DSBs signalling involves the phosphorylation of the histone H2AX (Ser139) and the protein p53 (Ser15). The various inhibitors of these DNA repair pathways (NU-7026, NU-7441 for DNA-PK; KU-55933 for ATM; AZ20 for ATR; and B02 for RAD51) are highlighted, along with the FWJ intermediate of the HR. Figure adapted from (156).

Although the central position of HJs in HR has been firmly established for decades (91), it is only recently that they were visualized in cells (124). While HR is only one of two major pathways leading to double strand DNA break (DSB) repair in mammalian cells, along with non-homologous end joining (NHEJ) (Figure 6) (125), it is indispensable in lower organisms (bacteria, yeasts) for ensuring DNA transfer and adapting to evolution (natural selection) (126). It is therefore unsurprising that a vast body of research has been dedicated to understanding the roles of HJ/FWJ in yeasts and bacteria.

These efforts have led to the discovery of the RuvABC complex that operates at HJs in prokaryotes (*Escherichia coli*) in a sequential manner: RuvA binds to HJs and targets RuvB to the junction, both RuvA and RuvB (RuvAB) promote branch migration and RuvC resolves the junction (127). An engineered synthetic protein RuvCDefGFP was recently used to investigate HJs in cells (128). RuvCDefGFP is a catalytically defective (Def) RuvC fused to green fluorescent protein (GFP); it was used to both map HJs in *E. coli* by HJ-ChIP-seq (using the anti-RuvC antibody), which demonstrated the accumulation of HJs near DSB sites, and visualize HJs *via* live-cell imaging, with a distribution correlated with homology-directed DSB repair, notably at single-stranded gaps.

These compelling results thus lend further credence to value of targeting HJs in bacteria by small molecules. The short hexapeptides developed by Anca M. Segall and coworkers were among the first reported HJ-ligands. It was first demonstrated that WKHYNY (Trp-Lys-His-Tyr-Asn-Tyr) target HJs *in vitro* and inhibits its resolution by the recombination proteins integration host factor (IHF, a heterodimeric protein that binds to and bends DNA) and the excisionase (Xis) (129). Next, it was shown by X-ray crystallography that WKHYNY does indeed bind to the central cavity of HJs (loxP sequence) (130) when a recombinase Cre tetramer maintains the HJ in its open form (110). The exact position of the peptide was not accurately determined, but found to be distributed over two major positions located on both sides of the C2-symmetrical cavity. Interestingly, the presence of the peptide modifies the structural organization of the HJ, which makes it less prone to be enzymatically resolved, thus providing some interesting insights into the mechanism by which hexapeptides inhibit HJ resolution.

This blocking behaviour was further investigated with two other peptides, WRWYCR (Trp-Arg-Trp-Tyr-Cys-Arg, Figure 3B) and KWWCRW (Lys-Trp-Trp-Cys-Arg-Trp), whose ability to inhibit both RecG helicase (which binds to and unwinds forked DNA) and RuvABC resolvase (which displays DNA junction specific helicase-endonuclease activity) in *E. coli* (131) was scrutinised (132). Their efficiency was quite high (e.g. with IC_50_ values >100 μM for WKHYNY versus 5–20 nM for both WRWYCR and KWWCRW against RecG; 0.06 μM for WRWYCR against RuvABC) but the exact mechanism remains unclear, as peptides do not prevent RuvA tetramer from binding to the HJ, RuvB does not interact with the central cavity of HJ *per se* and the cleavage activity of RuvC alone is marginally affected by the presence of the ligand. However, the peptides inhibit the resolvase activity of RuvC when embedded in the RuvABC complex, probably since this complex opens wide the cavity and makes it accessible to the peptides, which then blocks its enzymatic resolution for steric reasons. The most active peptide, WRWYCR, was indeed found more–and very–active against Gram^+^ bacteria (e.g. *S. aureus*, with minimal inhibitory concentration (MIC) between 4 and 32 μg/ml) than against Gram^–^ bacteria (e.g. *E. coli*, MIC between 32 and 64 μg/mL) (133). Of note, the presence of a cysteine in the most potent peptides implies that their active form is the disulfide-linked dimer: this was first demonstrated by the loss of activity upon Ala scan or addition of reducing agents (dithiothreitol, or DTT) (134) and by the design and use of the disulfide-independent dodecamer WRWYRGGRYWRW, found to be quite active (135). A series of fluorescence investigations performed with 2-aminopurines (2-APs) located at different positions around the central cavity of the FWJ along with molecular modelling studies confirmed a binding mode based on two hexapeptides interacting on both sides of the FWJ cavity (136). Attempts to favour these interactions using cyclic hexa- and octa-peptides were made: while the synthetic challenge was addressed (137,138), the macrocyclopeptides were not found to be very biologically active.

Given the central position of HJs in HR, it is unsurprising that HJ-trapping peptides have also been studied for their ability to impair recombination-based DNA repair, in both prokaryotes and eukaryotes. This was first scrutinized in bacteria, and it was found that the two most potent inhibitors (WRWYCR and KWWCRW), which bind to free HJs, display broad-spectrum bactericidal activity, notably against Gram^+^ bacteria (while the less potent WKHYNY, which binds to protein-bound HJs only, is not active) (133). The treatment of bacteria with peptides triggered DNA filamentation and segregation abnormalities, along with DNA break accumulation, in line with what is observed in cells undergoing severe DNA damage. The synergistic effects observed between peptide treatments and DNA damaging events (incubation with the cross-linking agent mitomycin C, or upon UV irradiation) show that DNA damage create more targets to which peptides bind (DNA lesions and hence repair); this delineates a novel antibacterial strategy based on HJ-trapping-mediated inhibition of recombination-dependent DNA repair. These studies were then extended to eukaryotic cells, particularly to human tumour cells (e.g. cervical cancer HeLa cells, prostate cancer PC3 cells) (139). The ability of WRWYCR to trigger DNA damage was evidenced by the immunodetection of both the histone H2AX phosphorylated on its serine 139 (γH2AX) (140) (Figure 6) and p53-binding protein 1 (53BP1), well-established markers of DSBs (141), along with the terminal dUTP transferase nick-end labelling (TUNEL) assay, likely due to the accumulation of unresolved DNA repair intermediates that leads to DNA breaks. Their toxicity was also potentiated by DNA damaging agents such as doxorubicin and etoposide, thus offering a new application to HJ-targeting agents as potential chemotherapeutics (either as standalone agents or in synthetic lethality cocktails). However, these studies were limited by the lack of information regarding the actual cellular specificity of these peptides for FWJs. Indeed, linear and cyclic short peptides are routine in medicinal chemistry (being used to fight against bacterial and viral infection, cancers and neuropathologies) (142), implying a broad variety of cellular targets. They also suffer from known limitations (conformational pluralism, limited bioavailability, short half-life *in vivo*, etc.) that, along with a low target selectivity, might limit and/or prevent their clinical use. None of these shortcomings were discussed here as these studies were intended to provide a proof-of-concept that targeting FWJs with small molecules can produce interesting cellular outcomes.

A way to tackle them is to use small molecules instead of peptides. To this end, Segall *et al.* also invested efforts to identify small molecules able to trap HJs and inhibit HJ resolution. Screening using the chemical libraries of the Torrey Pines Institute (TPI) led to the identification of a *N*-methyl aminocyclic thiourea referred to as TPI1530-1 (Figure 3B) as a potent inhibitor of RecG for instance (IC_50_ = 0.85 *versus* 0.12 μg/mL for WRWYCR). However, this compound was found inactive against bacterial growth, likely due to its poor bioavailability (143). A series of pyrrolidine bicyclic guanidine derivatives (TPI1609-1, -3, -10 and -12) were also identified, still *via* HTS-screens (144). Among them, TPI1609-10 displayed a still lower affinity for HJ than the hexapeptide WRWYCR (*K*_d_ = 300 versus 12.5 nM; but far higher than TPI1530-1, *K*_d_ = 11.8 μM), along with a lower RecG inhibitory activity (IC_50_ = 0.94 μg/ml), but elicited comparable, sometimes better MICs against Gram^+/–^ bacteria than WRWYCR (e.g. 16 versus 32–64 μg/ml against *E. coli*, respectively). These results thus provide strong support for the hypothesis that low-molecular weight molecules can indeed target HJs, inhibit their resolution and potently hamper bacterial growth. Unfortunately, no information is provided about the way these molecules interact with FWJs, and about their selectivity for FWJs versus other structures of DNA.

**Figure 7.**
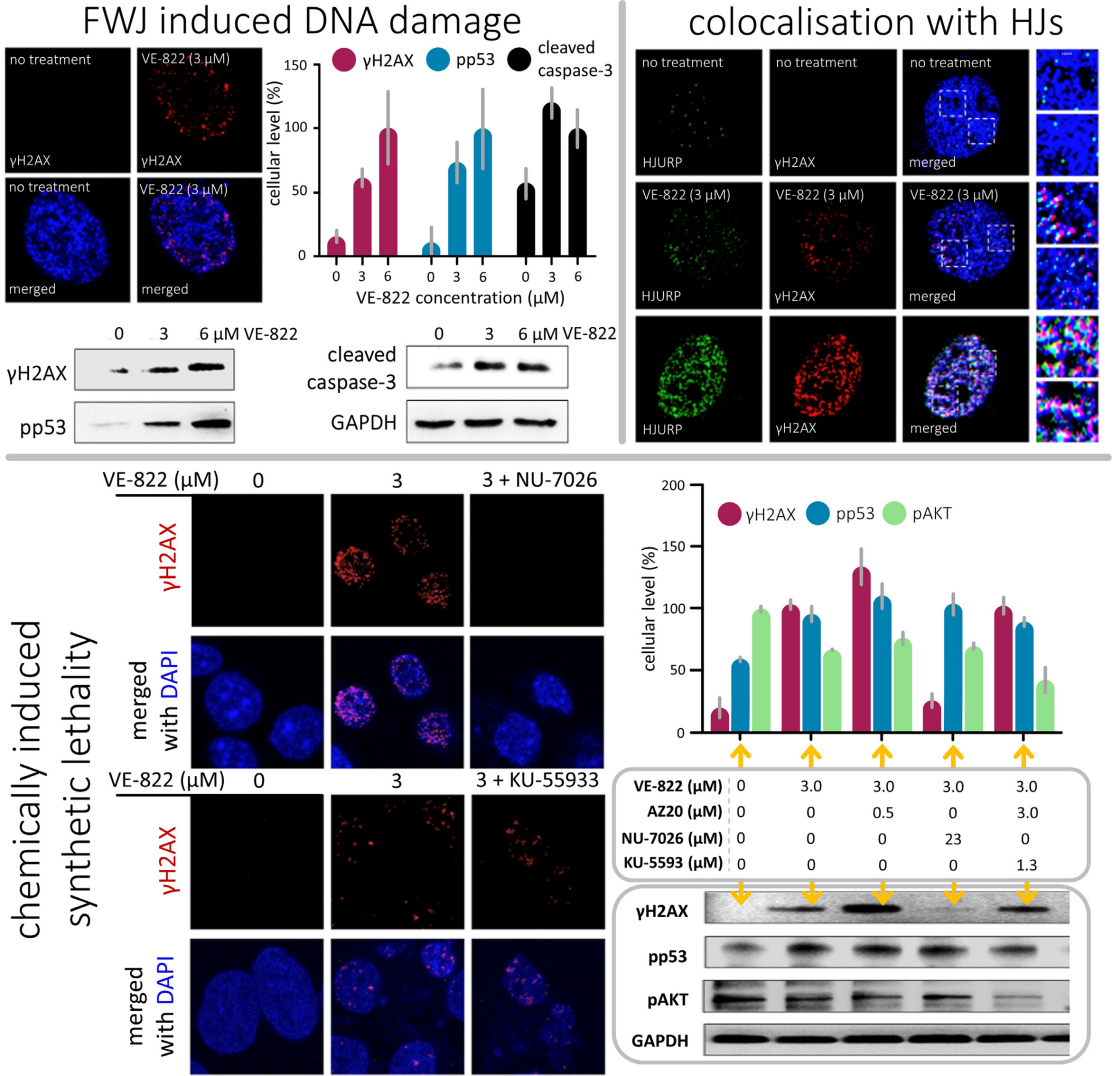
Cellular activity of the FWJ-ligand VE-822 that triggers extensive DNA damage (γH2AX and pp53 markers) eventually leading to apoptosis (cleaved caspase-3 marker), which colocalizes with Holliday junctions in U2OS cells (labelled using Holliday junction recognition protein, HJURP) and can be modulated by inhibitors of DNA repair (including AZ20, NU-7026 and KU-55933, which inhibit ATR, DNA-PKcs and ATM, respectively). Figure adapted from (146).

As indicated above, FWJ ligands have mostly been used to fight bacterial infections. But not only, with the DNA-damaging properties of WRWYCR also investigated in cancer PC3 and HeLa cells (139) and the antiproliferative activity of WRWYRGGRYWRW assessed in colorectal cancer HCT116 cells (135). To find a new FWJ-targeting scaffold, Searcey *et al.* developed an isothermal assay based on polyacrylamide gel electrophoresis (PAGE); this assay aimed at identifying small molecules able to trigger FWJ assembly from the four separated strands (119). It was initially implemented to screen a series of acridine-based ligands, whose chemical diversity was ensured by click chemistry (145); the resulting derivatives displayed only modest antiproliferative properties against human leukaemia HL60 cells, probably due to a poor cellular uptake, and no mechanistic rationale was provided about the origin of this cellular activity. Again, the demonstration of the specificity for FWJs of these molecules was lacking and their interaction (intercalation) with B-DNA not investigated; the authors also indicated a possible interaction with G4, thus complicating the interpretation of cell-based results. However, the goal of this study was clearly the development of a convenient *in vitro* assay to screen possible FWJ ligand candidates. It was incidentally implemented in an independent manner by Qikun Yin, Hongbo Wang and co-workers, which led to the discovery of a new FWJ-binding agent, VE-822 (Figure 3B) (146). This small molecule efficiently promotes FWJ assembly assessed by PAGE (EC_50_ = 7.6 μM) and confirmed by isothermal FRET assay (EC_50_ = 5.4 μM). It binds to FWJs with a high affinity (*K*_d_ = 8.6 μM) and a good selectivity over both dsDNA and ssDNA (*K*_d_ > 50 μM). In osteosarcoma (U2OS) cells, VE-822 was found to efficiently trigger DNA damage (γH2AX induction (140) along with phosphorylation of P53 on its serine 15 (pp53) (147,148), and of AKT on its serine 473 (pAKT) (149), Figures 6 and 7), which leads to cellular apoptosis (see the apoptosis marker cleaved caspase-3). DNA damage is mitigated by both the overexpression of the junction resolving enzyme BLM and cell pre-treatment with the DNA-PK inhibitor NU-7026 (150) (more modestly with ATR inhibitor AZ20 and ATM inhibitor KU-55933, Figures 6 and 7). These results highlight the role of DNA-PKcs (crucial for DSB signalling) (59) in VE-822-induced DNA damage sensing. To provide an unambiguous demonstration that DNA damage occurs at FWJ sites, co-immunodetection experiments were performed to colocalize γH2AX sites with the Holliday Junction Recognition Protein (HJURP, Figure 7) (151). Not only were common *foci* detected, but also their abundance was found to be modulated by either VE-822 (increase) or BLM overexpression (decrease), which thus establishes a direct link between ligand-stabilized FWJs and DNA damage. VE-822 is rather active against osteosarcoma (U2OS) and glioblastoma (U251) cells (IC_50_ ∼6 μM), more active than in healthy cells (e.g. lung fibroblast (WI38) and hepatocyte (HL7702) cells, with IC_50_ > 20 μM), which thus opens an interesting therapeutic window. Also, it synergistically interacts with the topoisomerase 2 (Top2) poison doxorubicin, an activity that can be modulated by either pre-incubation with NU-7026 or BLM over-expression (as above). Though comprehensive from a molecular and cellular biology point of view, this study suffers from the fact that VE-822 was initially developed as an ATR inhibitor (152). ATR is known, among other things, to prevent replication fork collapse and DSBs formation, and orchestrate DSB repair by the homologous recombination repair (HRR) pathway (153,154). Therefore, the inhibition of ATR by itself is expected to lead also to an accumulation of DNA damage markers (including γH2AX) and inhibit cancer cell growth, thereby casting doubt as to the actual origin(s) of the cellular effects observed here. However, this study is interesting in that it provides insights into FWJ biology beyond their role in DNA repair: the abnormal stabilisation of FWJs can indeed trigger DNA damage, likely as a result of the ability of FWJ/ligand complexes to hamper proper motion of polymerases along the genomic duplex. This role of roadblocking has been thoroughly studied in regards to TWJs (and will be thoroughly discussed below).

### Targeting three-way DNA junctions

In the examples described above, the interaction of small molecules with FWJs (or HJs) was intended to either impair DNA repair (their cellular activity being potentiated by DNA damaging agents such as doxorubicin) or trigger DNA damage (their cellular activity being modulated by DDR inhibitors). The situation is simpler with another class of branched DNA, the TWJs, as their stabilization is intended to trigger genetic instability by stabilizing physical obstacles that impair proper DNA-related enzymes processivity (such as polymerases and topoisomerases, involved in replication, repair and transcription). Indeed, junctions might arise ahead of the enzymatic complexes in charge of DNA transactions as a result of torsional stress originating in the motion of enzymes along the duplex. Stabilised TWJs might thus act as physical roadblocks to polymerases, which is recognized and coped with as a form of DNA damage by the cellular surveillance machinery (60–62). Therefore, the cellular activity of TWJ-targeting agents could be potentiated by DNA repair inhibitors (*vide infra*) (155,156).

The discovery of DNA junctions in general, and of TWJs in particular, was concomitant with the advances in electron microscopy in the early 1960s, which allowed for highlighting a series of branched DNA, although in most cases the strandness was not determined accurately. For instance, branched DNAs were visualized in the DNA of *Pneumococcus* (1961) (157), of both T4 (1962) (158) and T7 bacteriophages (1964) (159) and of *Xenopus laevis* oocytes (1968) (160). A hypothetical model for the formation of TWJ was postulated in 1964 by Arthur Kornberg (161), as part of his longstanding study on the biological synthesis of DNA. TWJs started to be studied in more detail when designed and assembled *in vitro* (162,163), to assess the specificity of the phage T4 endonuclease VII (162), or investigate the effect of unpaired nucleotides at the branch point on the overall stability of the junction (163). Naturally occurring TWJs have also been characterized in adeno-associated viruses (AAVs) (164). However, contrarily to FWJs, no central, constitutive roles were described for TWJs in eukaryotic cells. TWJs are thus plectonemic structures that can form transiently ahead of replication forks (RFs, *vide infra*), which can form structural obstacles that threaten genetic stability, in line with their genotoxic roles in repeat expansion diseases (165).

**Figure 8.**
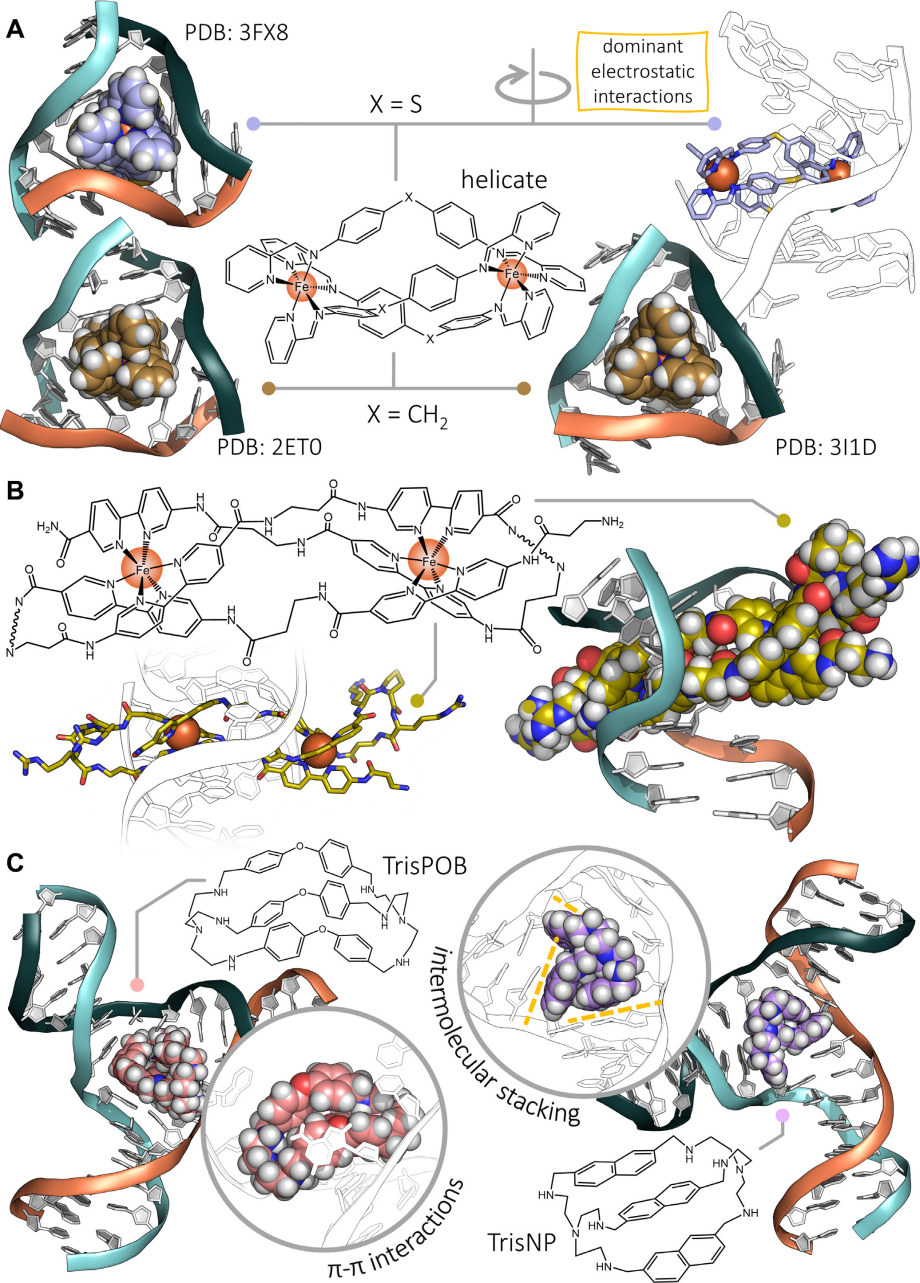
(**A**) X-ray crystal structures (PDB: 2ET0 (171), 3FX8 and 3I1D (172)) of a three-way DNA junction in interaction with dimetallic supramolecular cylinders (helicates). (**B**) Molecular dynamics (MD) simulation of a peptide helicate (Fe(II).LLD) in interaction with a TWJ (177). (**C**) Representative conformations of two azacryptands, TrisNP and TrisPOB, in interaction with a TWJ, obtained by MD simulations (156).

With this in mind, it is thus unsurprising that TWJs were targeted to create DNA damage according to a novel strategy, i.e. the indirect inhibition of polymerases *via* the stabilisation of physical obstacles they cannot cope with efficiently. Beyond the pioneering work of Kallenbach (with iron and copper complexes, e.g. MPE-Fe, Figure 3A) (89), the proof of concept that TWJs could be targeted by small molecules was incidentally provided by the design and use of aptamers as sensors for drugs (cocaine) (166–168) and steroids (e.g. cholic acid (169) and derivatives) (170). In these setups, TWJ-based aptamers were used firstly as turn-off (a dimeric TWJ is assembled upon small molecule binding), which brings the fluorophore (fluorescein) and the quencher (dabcyl) close to each other; (166,167) an externally added cyanine dye that is displaced from the cavity by the guest molecule) (168). Secondly, there are turn-on systems (a covalently linked fluorescein (quenched) that nestles within the TWJ cavity and is displaced by the guest molecule) (170). These results thus provided a basis for the design of hydrophobic TWJ ligands. The relevance of this approach was spectacularly demonstrated by Michael J. Hannon, Miquel Coll and coworkers with the elucidation of the X-ray structure of a TWJ/helicate complex (Figures 3C and 8A), in which the helicate (or triple-stranded dimetallic supramolecular cylinder) [Fe_2_L_3_]^4+^ triggers the assembly of a TWJ from a palindromic, 6-mer DNA sequence (171). This structure confirmed that the dimension of the cavity (ca. 1.1 nm diameter) makes it perfectly suited to accommodate rather large molecules (the size of the cylinder being ca. 2 nm length × 1 nm diameter). The exquisite recognition of TWJs by helicates was further demonstrated by another series of X-ray structure analyses (172), and confirmed by results collected *via* alternative techniques, including PAGE (173) and NMR (174). These results thus gave strong impetus to the use of sterically demanding small molecules, displaying shapes and volumes suited to ‘fill’ the TWJ cavity, including helical metallopeptides (studied by circular dichroism (CD), NMR and PAGE) (175–177), polyazamacrocyles such as TACN-Q (Figure 3C, by CD, PAGE, UV- and fluorescence resonance energy transfer (FRET)-melting assays) (178,179), porphyrins (by UV- and FRET-melting assays) (180), triptycenes (Figure 3C, by UV-melting and fluorescence quenching assays) (181,182), azacryptands such as TrisNP (Figure 3C) (155,156,179) and azacyclophanes (183,184) (by PAGE, FRET-melting and fluorescence quenching assays, and electrospray ionization mass spectrometry (ESI-MS) investigations), metallocages (by fluorescence titrations, fluorescence quenching assays and PAGE) (183–185), and calix[3]carbazoles (Figure 3C, by fluorescence titrations, CD, UV-melting assay and PAGE) (186).

### The relevance of TWJ in cells

With this series of fully characterised molecular tools in hand, cellular studies and chemical biology investigations were made possible, notably to assess the prevalence of TWJs in the human genome and the strategic relevance of targeting TWJs for creating DNA damage. The antiproliferative activity against human cancer cells were profiled for most of the aforementioned ligands (e.g. ovarian cancer SKOV3 cells and leukaemia HL-60 cells (187), melanoma B16 cells (179), breast cancer MCF-7 and MDA-MB-231 cells (184,188), colon carcinoma HCT-116 cells (189), etc.), but the mechanistic basis for this activity was sparsely established. These investigations were in fact slow to begin as a result of a combination of several factors: first, the biological relevance of TWJs in the studies performed with the first validated TWJ ligand (the dimetallic supramolecular cylinder [Fe_2_L_3_]^4+^) was misleadingly ascribed to RFs (171,187), while the fine structural details of RFs indicate that it is indeed a Y-shaped structure but made of unpaired sequences at the junction, unlike TWJs (190). Second, these first cellular investigations failed in providing convincing results, and the ability of [Fe_2_L_3_]^4+^ to interact with DNA in cells was even questioned (189). Third, the very nature of [Fe_2_L_3_]^4+^ made these investigations irrelevant owing to the ability of this supramolecular complex to interact with other DNA structures including the major grooves of B-DNA (191) and G4s (192,193). Therefore, this prototype was invaluable to uncover and accurately characterize a completely new DNA binding mode (with a molecule nestling within the central cavity of a TWJ) but was not ideally suited for chemical biology investigations. A very interesting observation, however, was that the closely related cylinder [Ru_2_L_3_]^4+^ was able to inhibit DNA transactions by preventing polymerase processivity; (194) this laid the basis for investigating whether TWJs might fold during DNA transactions in cells, more particularly at replication sites in the nucleus. To this end, co-localization studies were performed with a fluorescently labelled Fe(II) helicate (TAMRA-LLD, Figure 8B) and PCNA (proliferating cell nuclear antigen, a component of the replication machinery) fused to the green fluorescent protein (GFP) in HeLa cells (177). The collected images showed well-defined common *foci*, indicating that TWJs might fold transiently during DNA replication, in the vicinity of the replisome, and be trapped by *ad hoc* ligands. Similar co-localization studies were recently performed with a TWJ probe comprising a central, C3-symmetrical fluorogenic template (1,3,5-tristyrylbenzene) surrounded by three peptidic arms designed to interact with the three duplex arms of the TWJs (AT-hooks), thus found to preferentially accumulate in permeabilized cells where PCNA-GFP accumulates (195). However, the relevance of these results needs to be substantiated by a clear demonstration of the selectivity of used ligands for TWJs, as off-target effects cannot be ruled out on the basis of the currently available *in vitro* data.

**Figure 9.**
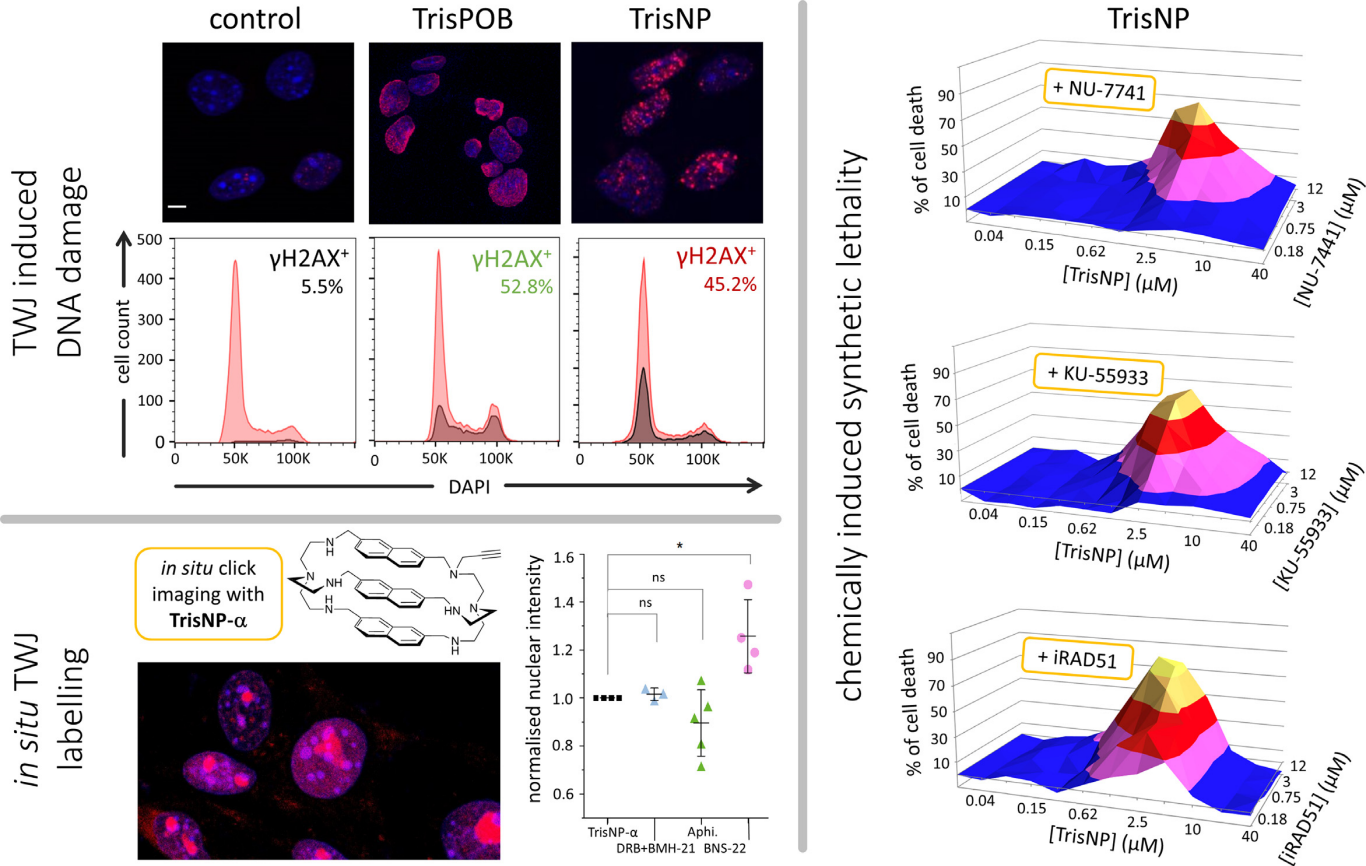
Cellular activity of the TWJ-ligands TrisNP and TrisPOB, which triggers extensive DNA damage in MCF7 cells (immunodetection of γH2AX and flow cytometry) that can be potentiated by co-incubation with inhibitors of DNA repair (including NU-7441, KU-55933 and B02, which inhibit DNA-PK, ATM and RAD51, respectively) (155). The number of TWJ *foci* labelled by *in situ* click chemistry using TrisNP-α as TWJ-ligand is not modulated by transcription inhibitors DRB and BMH-21, weakly modulated (decrease) by the replication inhibitor aphidicolin and strongly modulated (increase) by the Top2 inhibitor BNS-22 (156).

Studies were also performed to investigate whether TWJ ligands trigger DNA damage in dividing cells. This was firmly established *via* the immunodetection of γH2AX by both optical imaging and flow cytometry: after a thorough characterization of their TWJ-interacting properties *in vitro* (affinity and specificity *via* a panel of different techniques), two azacryptands (TrisPOB, TrisNP) were studied in cells and shown to trigger an accumulation of DNA strand breaks (and particularly DSBs) (Figures 3C, 8C and 9) (155,156). When combined with the established ability of TWJ ligands to prevent polymerase activity *in vitro* (194), these results thus provided a strong rationale for a mechanistic model in which ligand-stabilized TWJs act as roadblocks that hamper proper processivity of DNA-related enzymes, slowing or even stopping their motion along the genomic duplex, which is recognized as a DNA damage and trigger the DDR and repair machineries. We further exploited this model by using drug combinations in which TWJ ligands were used to induce DNA damage and DNA repair inhibitors to act synergistically with them, in an approach referred to as chemically induced synthetic lethality (196). In eukaryotic cells, DSBs can be repaired by either HR (*vide supra*) or non-homologous end joining (NHEJ) (Figure 6). The former relies on a broad array of protein effectors including RAD51 (which binds to single-stranded DNA, searches for sequence homology and favours strand exchange), and the kinases ATM and ATR (which bind to DSBs and stalled RFs, respectively); the latter also involves multiple processing enzymes, including kinases and ligases (e.g. DNA-PK and Lig4, respectively) (58,59,125). The antiproliferative activity of the TWJ ligands TrisPOB and TrisNP was found to be quite efficiently potentiated when combined with inhibitors of DNA-PK (NU-7441), ATM (KU-55933) and RAD51 (B02) (155,156), confirming that the cellular activity of TWJ ligands relies on the induction of strongly genotoxic DSBs (Figure 9). Interestingly, a synergy was also obtained with the Top2 inhibitor BNS-22: Top2 proteins resolve DNA topological stress (197) but are also involved in the recognition of alternative DNA structures and the formation of DSBs at these sites (198). Top2 also participates in the cleavage of hairpin structures formed from α-satellite sequences *in vitro* (199), which are centromeric regions known to be highly repetitive (200) and to fold into secondary structures (201). This synergy, confirmed by cytotoxicity assessments and γH2AX labelling, thus implies that Top2 inhibition favours TWJ formation, which was further demonstrated by bioorthogonal chemistry using the *in situ* clickable TWJ ligand TrisNP-α, Figure 3C) (156), which highlight the increase of the TWJ landscape (TrisNP-α labelling) upon Top2 inhibition (Figure 9). This opens brand new therapeutic opportunities, notably to treat cancers resistant to Top2 poisons.

As above, though comprehensive from a molecular and cellular biology point of view, these studies suffer from the fact that used TWJ ligands display a non-neglectable affinity for G4s. It was demonstrated *in vitro* that they display a *preferential* affinity for TWJ (i.e. in competitive setups where both TWJ and G4 are mixed, the ligands interact solely with TWJ) but this selectivity was not satisfying enough for an unambiguous interpretation of cellular outcomes. To confirm this interpretation, we showed by optical imaging that there is no overlaps between TWJ sites labelled with TrisNP-α (*in situ* click chemistry (202) with AF594-azide) and G4 sites labelled with the G4-specific antibody BG4 (35). These results are highly convincing but they are still not strong enough to unequivocally dispel doubts about the actual origins of the cellular effects monitored upon cell incubation with *bona fide* TWJ ligands. What the field now needs is a truly specific TWJ ligand; in light of the wealth of promising data described above, we can wager that a substantial research effort is currently being invested to identify the impatiently awaited game-changing TWJ ligand.

## CONCLUSION AND FUTURE DIRECTIONS

Targeting higher-order DNA structures with *ad hoc* ligands for chemical biology and/or medicinal chemistry purposes is now a commonly accepted strategy, undoubtedly as a consequence of the wealth of data accumulated about G4-DNA (68,69), the first-in-class example of a biologically relevant alternative DNA structure. While DNA junctions were discovered concomitantly with G4s (as indicated above, their basic structural unit was solved in 1962 (23) but their biological relevance was only discussed in the late 1980s) (24,203,204), the investigations aiming at confirming the existence of DNA junctions in human cells, scrutinising their functional relevance and establishing the reliability of the therapeutic strategies based on their targeting with small molecules still lag way behind that of G4s. This might be attributed to several factors: first and foremost, right after the pioneering report by Stephen Neidle, Lawrence H. Hurley and co-workers on a small molecule able to interact with a G4 (in the aim of indirectly inhibiting telomerase *via* the sequestration of its telomeric substrate under a form that is not recognized by the enzyme) (205), hundreds of G4 ligands have been synthesized and studied (75,206). This incredible enthusiasm has provided a strong impetus for the discovery of truly specific ligands (e.g. PhenDC3 (207), PDS) (208) which have soon become invaluable molecular tools to decipher G4 biology in a highly accurate manner (209). The DNA junction field has not experienced such a keen interest, presumably because the biological relevance of DNA junctions is still poorly understood. Also, the search for genomic G4 sites *via in silico* techniques (e.g. QuadParser (210), G4hunter (211), etc.) was straightforward thanks to the very nature of G4-forming sequences (example of used algorithm: d(G_3+_N_1–7_G_3+_N_1–7_G_3+_N_1–7_G_3+_)) (212). They have provided strong arguments about the prevalence of G4s in the human genome, which was soon connected to a widespread functional relevance. The nature of the DNA junction-forming sequences (direct or inverted sequence repeats, and not easily identifiable motif repeats such as GGG triplets) make them more difficult to be reliably predicted at a genome-wide scale (although some laudable attempts have been made, such as IRfinder (213), palindrome analyzer (214), etc.). Again, this has contributed to somewhat dampen enthusiasm for the search of genomic DNA junction-forming sites. Finally, the challenge of identifying genomic G4 sites by ChIP-seq-like techniques (such as G4 ChIP-seq) (50) was met, thanks to the development of the G4-specific antibody BG4 (35), and no similar antibody exists in the DNA junction field, which makes both their identification (ChIP) and visualisation (immunofluorescence) challenging and regularly questioned.

Despite these difficulties, the aforementioned efforts have started to pay off, but the prevalence of putative DNA junction-forming sequences described above (37,41) combined with the lack of fine details regarding DNA junction biology (which originates in the lack of reliable molecular tools) explain why this chemical biology quest is still in its infancy. Massive efforts must now be invested to keep on developing tools and technologies to interrogate and manipulate DNA junctions in a functional cellular context. The next steps will thus be to (i) identify truly specific ligands for DNA junctions, in order to establish a reliable link between junction interaction in cells and the cellular outcomes monitored; (ii) create and/or optimize *in vitro* screening assays to blind screen commercially available chemolibraries in order to expand the portfolio of DNA junction ligand candidates far beyond rational design; (iii) develop on the basis of identified promising scaffolds multivalent molecular tools (bearing for instance an alkyne appendage that could be manipulated in a bioorthogonal manner in cells) to identify the sequences they interact with (direct repeats? inverted repeats? other?) and the proteins associated combining both sequencing (bioinformatic tools will have to be optimized as well) and proteomics. Of note, the identification of DNA junction-binding proteins will be a very important step as they are the cellular effectors by which the response to junction stabilization by *ad hoc* ligands is mediated; while hundreds of G4-binding proteins are now known (215), only a handful of junction-binding proteins have been reported to date (e.g. RuvABC and RecG, *vide supra*); (iv) produce DNA junction-specific antibodies for both immunoprecipitation and immunodetection purposes, and (v) develop cellular and small animal models to assess the properties of identified candidates in a standardized manner.

The ultimate goal of these investigations will be to rapidly provide a proof-of-concept that DNA junction-targeting molecules can be considered as key players in the field of therapeutic agents, to validate all the necessary pre-clinical milestones in a reliable manner in the aim of reaching the clinical stage rapidly and confidently. Without a doubt, these chemical biology investigations will lead to major advances in research on this new class of therapeutic targets, a momentum that will contribute to both better understand the biology of DNA junctions (which will find applications in diverse therapeutic areas such as cancers (62), neuropathologies (165) and infectious diseases) and unravel the fascinating structural and functional diversity of DNA.

## DATA AVAILABILITY

No new data were generated or analysed in support of this research.
