# Correction to ‘Interactions of small molecules with DNA junctions’

**DOI:** 10.1093/nar/gkac1161

**Published:** 2022-12-09

*Nucleic Acids Research*, 2022, gkac1043, https://doi.org/10.1093/nar/gkac1043

In the Abstract and Introduction, base A has been mistakenly paired with C instead of T. The following sentences have been corrected:

In the Abstract:

The four natural DNA bases (A, T, G and C) associate in base pairs (A = C and G≡C), allowing the attached DNA strands to assemble into the canonical double helix of DNA (or duplex-DNA, also known as B-DNA).

has been corrected to

The four natural DNA bases (A, T, G and C) associate in base pairs (A = **T** and G≡C), allowing the attached DNA strands to assemble into the canonical double helix of DNA (or duplex-DNA, also known as B-DNA).

In the Introduction:

The topological diversity of DNA stems from supramolecular chemistry considerations: nucleobases (A, C, G, T) associate through the formation of hydrogen bonds (H-bonds), two in the A = C base pair, three in the G≡C base pair, allowing for a dynamic assembly/disassembly without substantial energy penalty.

has been corrected to

The topological diversity of DNA stems from supramolecular chemistry considerations: nucleobases (A, C, G, T) associate through the formation of hydrogen bonds (H-bonds), two in the A = **T** base pair, three in the G≡C base pair, allowing for a dynamic assembly/disassembly without substantial energy penalty.

In addition, a source of Funding has been updated:

Agence Nationale de la Recherche (ANR-22 InJUNCTION, D.M./A.P.)

has been corrected to

Agence Nationale de la Recherche (**ANR-22-CE44-0039**, D.M./A.P.)

